# Survival of *Escherichia coli* O157:H7 in Soils Along a Natural pH Gradient

**DOI:** 10.3390/microorganisms13112492

**Published:** 2025-10-30

**Authors:** Guangze Lyu, Huiru Li, Jiayang Hu, Jincai Ma

**Affiliations:** 1 Key Laboratory of Ground Water Resource and Environment, Ministry of Education, Jilin University, Changchun 130021, China; lvgz19@mails.jlu.edu.cn; 2 Jilin Provincial Key Laboratory of Water Resources and Environment, Jilin University, Changchun 130021, China; lihr2016@foxmail.com (H.L.); hjy23@mails.jlu.edu.cn (J.H.)

**Keywords:** *E. coli* O157:H7, survival, pH gradient, soil, bacteria community, structural equation model

## Abstract

*Escherichia coli* O157:H7 (EcO157) is a Gram-negative foodborne pathogen capable of transmitting between soil, food, and humans, posing a threat to human health. The soil pH in Jilin Province decreases gradually from west to east, exhibiting a natural pH gradient zone. Persistence of EcO157 in soils from different places was widely reported, while its survival behavior in soils over a pH gradient is yet to be investigated. In the current study, a total of 24 soil samples were collected along a natural pH gradient. Soils were classified into weak acidic soil (pH < 6.5), neutral soil (6.5 < pH < 7.5), weak basic soil (7.5 < pH < 8.5), and strong basic soil (8.5 < pH < 10). EcO157 cells were inoculated into those soils and the survival profiles were investigated. The influencing factors affecting the survival behavior of EcO157 were analyzed by multivariate statistical analysis. The results showed that the average survival time of EcO157 in weak acidic, neutral, weak basic, and strong basic soils was 61.08, 72.05, 76.85, and 18.54 days, respectively. The survival time in strong basic soils was significantly less than that in the other three soil groups. Soil physicochemical properties such as NO_3_^−^-N and NH_4_^+^-N were negatively linked to the survival of EcO157, while total phosphorus (TP)was positively correlated to the survival of EcO157 (*p* < 0.05). The microbial community α diversity index was negatively correlated with the survival of EcO157, while relative abundance of *Proteobacteria* and *Acidobacteria* was positively and negatively correlated to the survival of EcO157, respectively. Both co-occurrence network analysis and structural equation model results showed that pH was a key factor that could directly and indirectly influence the survival of EcO157 via the bacterial community. Our data coupled with the findings of other studies might be of great help in the evaluation, control, and reduction of the potential health risk associated with EcO157 in soils along a natural pH gradient.

## 1. Introduction

*Escherichia coli* O157:H7 (EcO157) is a bacterial pathogen that can infect humans at a very low infectious dose [1]. Once infected by this pathogen, patients can potentially manifest a life-threatening symptom, i.e., hemolytic uremic syndrome [2]. EcO157 is generally associated closely with public health due to its foodborne and waterborne modes of transmission [3]. EcO157 cells can contaminate agricultural soils via irrigation with polluted water sources, or via organic fertilizer application with poorly composted manure [4]. Foodborne outbreaks, such as the ones that occurred in Japan and the United States, were thought to be caused by the radish sprout and bagged spinach and lettuce [5], respectively. The health risk of EcO157 is largely linked to its survival potential in the environment. EcO157 can survive in soils for a significant time, ranging from weeks to months [6]. Previous studies showed that EcO157 survived for about 30 days in soils from Salinas, USA and for 70 days in soils from Northeastern China [7].

Natural and anthropogenic activities could result in different environmental gradients, along which striking variation in the fate and transport of pollutants were reported, e.g., brominated flame retardants at altitude gradient [8], halogen at moisture gradient [9], and antibiotic resistance genes at slope gradient [10]. It was also documented that such environmental gradients also significantly affected the indigenous plant and microbial communities [11,12]. Recently, it was found that the soil pH gradually decreases from the west to east of Jilin Province, China [13], and obviously a pH gradient was observed. The soil pH of Baicheng City in western Jilin was significantly higher than that of other areas, with an average of 8.1 [13]. The soil pH of Changchun and Siping City in the central region of Jilin Province was at a neutral level, averaging at 6.5 and 6.8, respectively. The soil pH of Yanbian Prefecture in the eastern part of Jilin was obviously low, with an average pH of 5.6 [13,14]. The main reason for pH gradient formation was due to the reduction in annual mean temperature and precipitation from west to east of Jilin Province. Land use types may also contribute to the formation of pH gradients [15]. Since soil properties may vary at different pH values, and such changes will significantly affect the environmental behaviors of pollutants, including both chemical and biological contaminants, it will be interesting to investigate the fates of typical human pathogens, such as EcO157, in soils over such a pH gradient.

Both biotic and abiotic factors can exert influence on the survival of EcO157 in soils [16]. Previous research indicated that pH was one of the crucial abiotic factors influencing the survival of pathogens in soils and was positively correlated with the survival of EcO157 [17,18,19]. It has also been reported that it is more difficult for EcO157 to survive in sandy soils than in clay soils [10]. Higher salinities may result in shorter survival times for EcO157 in soils [14]. Organic carbon and nitrogen content in soils were positively correlated with the survival of EcO157 since they provide the necessary nutrients for pathogens [18,20]. On the other hand, it is well established that microbial diversity was negatively correlated with the survival of EcO157 [21]. As an invading pathogen, EcO157 may directly suffer due to microbial resistance in indigenous species. It has been certified that indigenous *Proteobacteria*, especially the subclass *γ-Proteobacteria*, could negatively affect the survival of EcO157 to a greater extent when compared with other soil bacterial groups, such as *Firmicutes* [22]. However, other indigenous bacterial groups, e.g., Actinobacteria and *Acidobacteria*, displayed a positive correlation to the survival of EcO157 [20]. The potential mechanisms for this occurrence might be explained by viewing EcO157 as a threat to the stability of the microbial environment; thus, it triggers subsequent changes in the composition and structure of bacterial communities [22]. However, there has been no research conducted to systematically investigate the interactions between EcO157 and soil properties in soils over a natural pH gradient [23,24,25].

In the current study, we collected and characterized soil samples along the pH gradient as mentioned above and the EcO157 cells were inoculated in those samples to test their survival potential. The objectives of this study were to (1) investigate the survival behavior of EcO157 in soils along a natural pH gradient and (2) identify key factors influencing the survival parameters. Our study aims to provide insights into the mechanisms of the survival behavior of EcO157 in soils with distinct pH values.

## 2. Materials and Methods

### 2.1. Soil Sample Collection and Characterization

**Figure 1:**
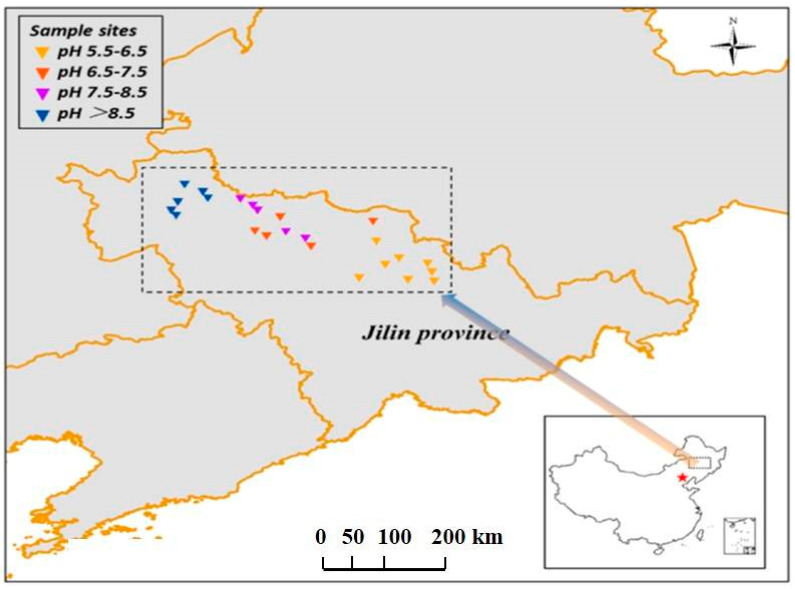
Distribution map of soil sampling sites. The varying color of triangles from blue to yellow represents soil pH range from high to low.

As shown in Figure 1, a total of 24 soil samples were collected, and those samples were almost evenly distributed in the western, central, and eastern parts of Jilin Province. The latitude of 24 soils was between 43°50′6″ and 45°38′12.14″, and the longitude was between 123°33′1″ and 127°19′34″ (Table S1). Top soils (0–20 cm) were sampled, and each sample was a composite of 3 individual soil cores spaced at 5 m intervals. The soil was mixed, homogenized, and sifted (<2 mm) to remove plant roots, debris, and stones. The samples were divided into two parts; one part was air-dried and used for the determination of physicochemical properties, and the other part was stored in a −80 °C refrigerator (Heli, Lanxi, China) for DNA extraction. Soil particle content was determined using a laser particle size analyzer (Bettersize, Dandong, China). A pH meter (Mettler, Shanghai, China) was used to determine soil pH. A digital conductivity meter (Rex, Shanghai, China) was applied to determine electrical conductivity salinity (EC, μS/cm, soil–water ratio of 1:2.5). Total phosphorus (TP, mg/kg), ammonium nitrogen (NH_4_^+^-N, mg/kg), and nitrate nitrogen (NO_3_^−^-N, mg/kg) were measured using a UV-Vis spectrophotometer (MapData, Shanghai, China). Water-soluble organic carbon (WSOC, mg/kg, soil–water = 1:2.5) was quantified using a TOC instrument (Shimadzu, Kyoto, Japan). The soil sample’s physicochemical properties are shown in Table S2.

### 2.2. Bacterial Strains

EcO157 EDL931 (ATCC 35150) was used in the soil survival assays. EDL931 was a human feces isolate conferring *stx*_1_, *stx*_2_, and *eas* genes [26]. In order to facilitate the enumeration of EDL931 on Sorbital MacConkey agar supplemented with BCIG (5-bromo-4-chloro-3-indoxyl-β-D-glucuronide), nalidixic acid (Nal, 25 μg/mL), and rifampicin (Rif, 100 μg/mL), the EDL931 wild-type was tagged with Nal (25 μg/mL) and Rif (100 μg/mL) resistance by selection of mutants grown on LB (Luria−Bertani) agar supplemented by Rif, and Nal + Rif sequentially. It was confirmed that the growth curve of the mutant in rich media (LB broth) was identical to that of the wild-type strain. In addition, the survival curve of the mutant in soils with different structures (sandy, loamy, and clayey) was found to be identical to that of the wild-type strain [27].

### 2.3. Survival of E. coli O157:H7 in Soils

Inoculum preparation and inoculation procedure were reported previously [7]. In brief, early stationary phase cells were used after washing (0.9% saline water) and starvation (1 h in the dark). The cells were inoculated into the soil to a final concentration of 5 × 10^6^ colony-forming units per gram of soil dry weight (CFU gdw^−1^). All experiments were conducted under room temperature (22 ± 1 °C). Triplicate plastic bags containing the soils inoculated with EcO157 were prepared. Moisture content of the soil sample was maintained constantly (60% of WHC) during the course of experiment by adding additional deionized water to make up for evaporation. Soils without EcO157 inoculation were used as a control. At days 0, 1, 2, 4, 6, 8, 11, 13, 15, 19, 25, 32, 37, 47, 57, 68, 80, and 96, the inoculated soils were sampled. Cells were extracted and plated on the selective agar plates, and finally, the CFU was counted according to the methods described previously [7].

### 2.4. Soil Bacterial Communities’ Characterization

The hypervariable V4 region of the 16S rRNA gene was amplified using the primers: 16S-F (5′-AYTGGGYDTAAAGNG-3′) and 16S-R (5′-TACNVGGGTATCTAATCC-3′) [28]. Sequencing was performed on an Illumina MiSeq instrument (Illumina, San Diego, USA) using a paired-end 150 bp sequence read run with the Miseq Reagent Kit v3 at the Personal Biotechnology Company (Shanghai, China). Each DNA sample was individually PCR-amplified in triplicated 25 μ L reactions. The cycling conditions included an initial denaturation at 94 °C for 5 min, followed by 25 cycles at 94 °C for 30 s, 50 °C for 30, 72 °C for 30 s, and a final 7 min extension at 72 °C. Each reaction contained 1 × PCR buffer, 2.5 mM dNTPs, 0.625 U of Taq DNA polymerase (Takara Bio, Otsu, Shiga, Japan), 10 μM of each primer, and 20 ng of template DNA.

Bacteriome bioinformatics were mainly performed with QIIME 2 [29] while the OTU clustering procedure following the Vsearch (v2.13.4) [30] pipeline described here “https://github.com/torognes/vsearch/wiki/VSEARCH-pipeline (accessed on 8 April 2024)”. Briefly, raw sequence data were demultiplexed using the demux plugin followed by primers cutting with cutadapt plugin [31]. Sequences were then merged, filtered, and dereplicated using functions of fastq_mergepairs, fastq_filter, and derep_fulllength in Vsearch. All the unique sequences were then clustered at 98% level (via cluster_size) followed by chimera removing (via uchime_denovo). Finally, the non-chimera sequences were re-clustered at 97% level to generate OTU representative sequences and OTU table. Representative sequences were aligned with mafft [32] and used to construct a phylogeny with fasttree2. Dominant OTUs were defined as those with a representation ≥ 5% within a sample; abundant OTUs were defined as those with a representation ≥ 1% within a sample; and rare OTUs were defined as having an abundance < 0.01% within a sample. The sequence data have been deposited to NCBI BioProject under accession number PRJNA530627.

### 2.5. Survival Data Modeling

The survival of EcO157 was analyzed by fitting the experimental data to the Weibull survival model using GInaFiT v1.5 [33,34]. The Weibull survival model was constructed based on the hypothesis that the deactivation kinetics of the EcO157 population followed a Weibull distribution [21]. The size of the surviving population could be calculated using Equation (1),

(1) $$\log\left(N_{t}\right)=\log\left(N_{0}\right)-{\left(t/\delta \right)}^{p}$$

where *N*_t_ is number of survivors, *N*_0_ is inoculum size, *t* is time (days) post-inoculation, *δ* is a scale parameter representing the time (days) needed for first decimal reduction, and *p* is a non-unit shape parameter. When *p* > 1, <1, and =1, a convex, concave, and linear curve was expected [35]. Time needed to reach detection limit (*ttd*) could also be calculated by modeling the data. The detection limit was 100 CFU gdw^−1^.

### 2.6. Statistics Analysis

Stepwise multiple regression analysis of *ttd* with soil physicochemical properties and bacterial community structure (Shannon–Wiener index (*H*), total OTU count, and the relative abundances of dominant phyla) was performed using SPSS v22.0 (IBM, Chicago, IL, USA). The α diversity indices (Chao1, ACE, and Shannon–Wiener) were calculated by R 3.5.2 with vegan package. β diversity was determined by non-metric multidimensional scaling (NMDS) and the first axis score of each sample was used to represent the community structure. Mantel and Partial Mantel Tests were used to establish the correlations among *ttd*, soil physicochemical properties, and bacterial community structure. NMDS, Mantel and Partial Mantel Tests were conducted by R 3.5.2 with vegan package. In order to clearly demonstrate the correlation between survival parameters, physicochemical properties, and bacterial communities, co-occurrence network analysis based on Spearman’s correlation (*p* < 0.05) was performed on a R 3.5.2 platform with Hmisc v4.6-0 and Igraph v1.2.11 packages and was visualized using Gephi “https://gephi.org/ (accessed on 16 July 2024)”. The survival parameters *ttd*, *p*, and *δ* formed a primary matrix, and the secondary matrix was composed of soil physicochemical properties and microbial community structure. Both matrices were subjected to canonical correlation analysis (CCA). Structural Equation Modeling (SEM) was performed using SPSS coupled with AMOS 20.0 (IBM, Chicago, IL, USA).

## 3. Results

### 3.1. Soil Characterization and Grouping

Results of soil characterization showed that soil pH was between 5.61 and 9.92. EC ranged from 0.04 to 8.57 mS/cm. NH_4_^+^-N varied from 0.55 to 40.79 mg/kg, while NO_3_^−^-N varied from 0.12 to 240.02 mg/kg, and total phosphorus was between 2.05 and 36.10. Clay was from 2.8 to 21.07 and WSOC was in a range of 26.65 to 2182.40 mg/kg (Table S2).

In order to highlight the differences between soil pH, we divided the soil samples into four groups according to their pH values. Soils with pH < 6.5 were classified as weak acidic soils, and included soil samples T1-T8; soils with 6.5 < pH < 7.5 were classified as neutral soils, and included soil samples T9-T13; soils with 7.5 < pH < 8.5 were classified as weak basic soils, and included soil samples T14-T18; and soils with pH > 8.5 were classified as strong basic soils, and included soil samples T19-T24.

### 3.2. Soil Bacterial Community Characterization

**Figure 2:**
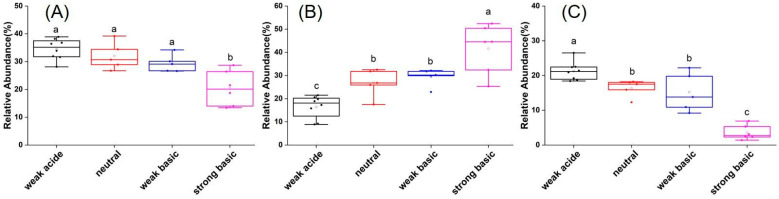
Relative abundances of *Proteobacteria* (**A**), *Actinobacteria* (**B**), and *Acidobacteria* (**C**) in soils with different pH ranges. Bars labeled with different letters are significantly different (*p* < 0.05).

From the high-throughput sequencing analysis, a total of 22022 OTUs were obtained from the 24 soil samples after normalization. *Proteobacteria*, *Actinobacteria, Acidobacteria*, *Chloroflexi*, and *Gemmatimonadetes* were the dominant phyla among the bacterial communities in all soil samples (Figures S1 and S2). At the class level, the dominant groups in all soil samples were *Alphaproteobacteria*, *Actinobacteria*, *Subgroup 6*, *Thermoleophilia*, *Betaproteobacteria*, *Gemmatimonadetes*, *Gammaproteobacteria*, *KD4-96*, *Acidimicrobiia*, *Deltaproteobacteria*, *Acidobacteria*, *Blastocatellia*, and *Solibacteres* (Figure S3). Further analysis showed that in weak acidic soils, *Proteobacteria* was the most abundant phyla and the relative abundance of *Acidobacteria* was higher than in the other three soils. Instead, *Actinobacteria* was the most abundant phyla and *Acidobacteria* was the least abundant phyla in strong basic soils. In neutral and weak basic soils, the abundance of major phyla was familiar, exhibiting that *Proteobacteria* and *Actinobacteria* were the two abundant phyla (Figure 2). The results indicated that all soil samples exhibited the same trend in α-diversity indices, as reflected in Chao1, ACE, and Shannon-Wiener index (Table S3). The diversity indices of the strong alkaline soil group were the lowest when compared with the other soil groups. Results of NMDS analysis (β diversity) showed that the soil sample points in each group were well separated from each other, and such a trend was more pronounced for strong alkaline soil samples (Figure S4, Table S4).

### 3.3. Survival Behavior of E. coli O157:H7 in Soils

**Figure 3:**
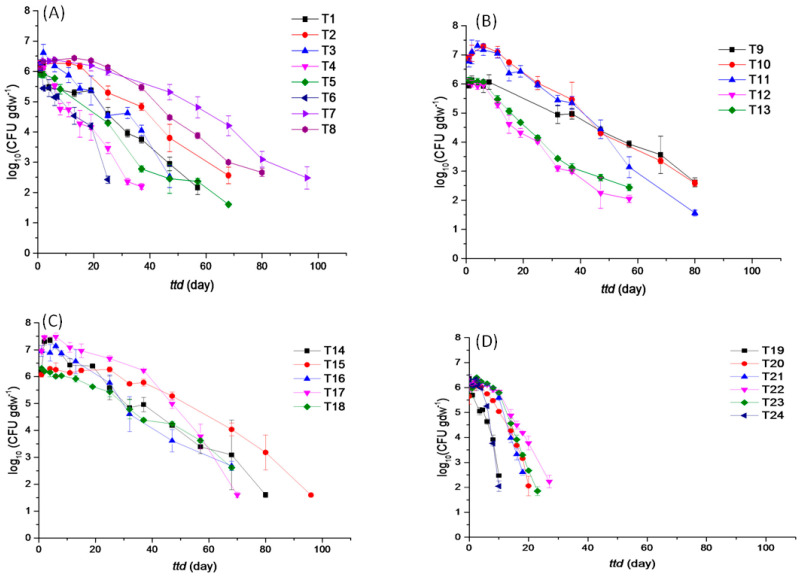
Survival curves of *E. coli* O157 in weak acidic soils (T1–T8) (**A**), neutral soils (T9–T13) (**B**), weak basic soils (T14–T18) (**C**), and strong basic soils (T19–T24) (**D**).

**Figure 4:**
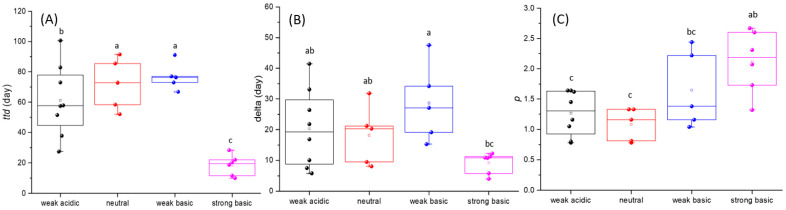
Time needed to reach detection limit (*ttd*) (**A**); the first decimal reduction time (*δ*) (**B**); and the shape parameter (*p*) (**C**) of weak acidic, neutral, weak basic and strong basic soils. Error bars represent the standard deviation of the triplicate measures of *ttd*, *δ*, and *p*. Bars labeled with different letters are significantly different (*p* < 0.05).

Survival profiles of EcO157 in soils are presented in Figure 3. It shows that all survival curves display convex shapes, which is consistent with the finding that survival time is shortest in strongly alkaline soils when the *p*-value is greater than 1. When 24 soil samples were taken into account, average *ttd* was 56.02 days and ranged from 9.99 to 100.70 days (Figure 4A). Weak acidic soils had an average *ttd* of 61.08 days, ranging from 27.40 to 100.70 days. Neutral soils had an average *ttd* of 72.05 days, ranging from 52.00 to 92.50 days. Weak basic soils had an average *ttd* of 76.85 days, ranging from 66.85 to 91.10 days. Strong basic soils had an average *ttd* of 18.54 days, ranging from 9.99 to 28.40 days.

The *δ* value had an average of 18.83 days, ranging from 4.00 to 47.54 days in all the soil samples (Figure 4B). Weak acidic soils had an average *δ* value of 20.36 days, ranging from 5.79 to 41.46 days. Neutral soils had an average *δ* value of 18.18 days, ranging from 8.05 to 31.84 days. Weak basic soils had an average *δ* value of 28.63 days, ranging from 15.24 to 47.54 days. Strong basic soils had an average *δ* value of 9.16 days, ranging from 4.00 to 12.24 days.

Among the 24 soil samples, *p* had an average value of 1.52, ranging from 0.78 to 2.67 (Figure 4C). Weak acidic soils had an average *p* of 1.27, ranging from 0.78 to 1.64. Neutral soils had an average *p* of 1.08, ranging from 0.78 to 1.33. Weak basic soils had an average *p* of 1.65, ranging from 1.04 to 2.44. Strong basic soils had an average *p* of 2.12, ranging from 1.32 to 2.67.

The *ttd* of weak basic soils was the longest, followed by neutral, weak acidic, and strong basic soils. Correspondingly, the *δ* value of the neutral group was the largest, followed by neutral, weak acidic, and strong basic soils. As for *p*, there was no significant difference (*p* < 0.05) among weak acidic soils, neutral soils, and weak basic soils.

### 3.4. Stepwise Multiple Regression, Mantel and Partial Mantel Tests Analysis

The stepwise multiple regression equation shows that pH, NO_3_^−^-N, *Proteobacteria*, *Acidobacteria*, and *H* were major factors influencing the survival of EcO157 in soils (Table 1). To be more specific, pH, NO_3_^−^-N, and *Acidobacteria* are negatively correlated with *ttd* and *Proteobacteria*; *H* is positively correlated with *ttd*.

To further discuss the effects of various factors on *ttd*, Mantel and Partial Mantel tests were conducted. The results of the Mantel test showed that *H*, bacterial community, NO_3_^−^-N, pH, *Acidobacteria*, WSOC, EC, *Proteobacteria*, NH_4_^+^-N, and *Actinobacteria* were all correlated with *ttd* (Table 2). We also carried out a Partial Mantel test to determine the net effect of each factor on *ttd*; the results showed that *H*, bacterial community, NO_3_^−^-N, and pH were still correlated with *ttd* after partialing out other factors.

### 3.5. Co-Occurrence Network of Survival Time, Soil Properties, and Microbial Community Composition and Structure

**Figure 5:**
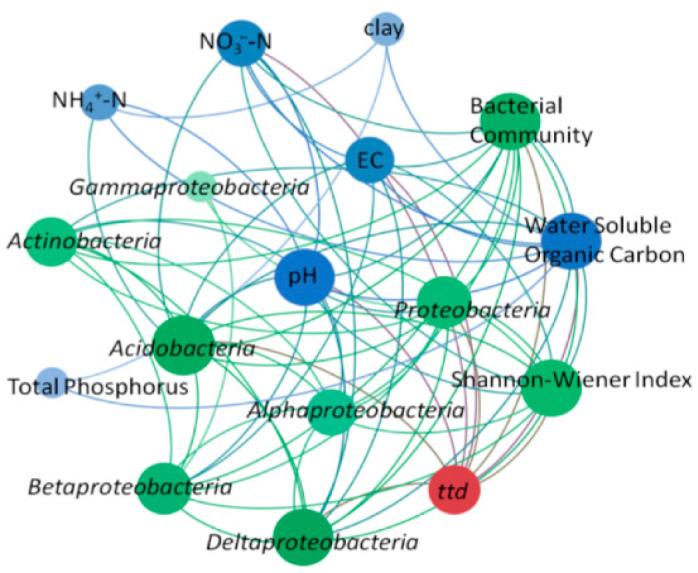
Co-occurrence network showing the interaction between *ttd*, soil physicochemical properties, and bacterial communities. The parameters directly connected with *ttd* suggest that they had a direct impact on *ttd*. Red dots indicate the survival parameter *ttd*, blue dots indicate abiotic factors, and green dots indicate biotic factors. The darker the parameter, the more of a connection it has with other factors, such as WSOC, pH, NO_3_^−^-N, *Acidobacteria*, and the bacterial community. The lighter parameters, such as TP, clay, and *δ-Proteobacteria*, are less associated with other factors. EC, electrical conductivity; NH_4_^+^-N, ammonium nitrogen; NO_3_^−^-N, nitrate nitrogen; TP, total phosphorus; bacterial community (the first axis scores from non-metric multidimensional scaling); pH, potential of hydrogen.

Co-occurrence network analysis was used to provide a visual depiction of the factors affecting *ttd*, including abiotic factors such as various soil physicochemical properties (pH, WSOC, EC, NO_3_^−^-N, NH_4_^+^-N, clay, TP) and biotic factors such as the structure of the bacterial community, relative abundances of *Proteobacteria*, *Acidobacteria*, *Actinobacteria*, *α-Proteobacteria*, *β-Proteobacteria*, *γ-Proteobacteria*, and *δ-Proteobacteria* (Figure 5). In Figure 5, red dots represent *ttd*, blue dots indicate soil physicochemical properties affecting EcO157 survival, and green dots represent biological factors. The larger the circle and the darker the color, the more significant the effect of the factors on *ttd*. The key topological parameters of the co-occurrence network are presented in Table S5. The major influencing factors included pH, WSOC, NO_3_^−^-N, bacterial community, *H*, *Proteobacteria*, and *Acidobacteria*. The influencing factors obtained from CCA completely overlap with the results of the co-occurrence network analysis (Figure S5).

### 3.6. Structural Equation Model of Survival Data, Soil Properties, and Bacterial Community

**Figure 6:**
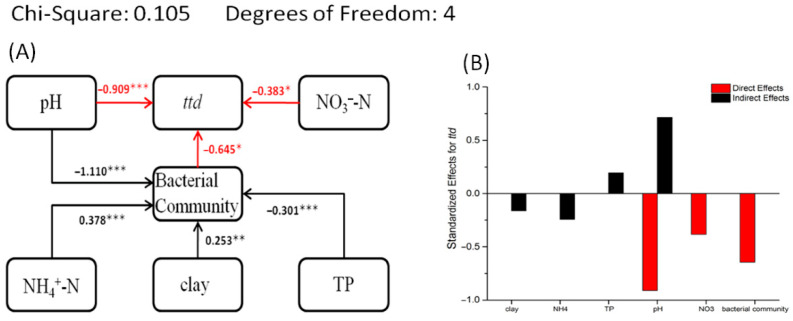
Structural equation models (SEMs) based on direct and indirect effect of soil properties and bacterial community structures on the survival parameters *ttd* (**A**) of *E. coli* O157:H7. The direct and indirect effects of environmental factors on *ttd* are quantified (**B**). ***, **, and * indicate that *p* values are significant at 0.001, 0.01, and 0.05 levels, respectively. The red arrow indicates a direct impact on the survival parameter *ttd*. NH_4_^+^-N, ammonium nitrogen; NO_3_^−^-N, nitrate nitrogen; TP, total phosphorus; bacterial community, the first axis scores from non-metric multidimensional scaling; pH, potential of hydrogen.

The results of SEM show (Figure 6) that NO_3_^−^-N and bacterial community structure (the first axis scores from non-metric multidimensional scaling) has a direct effect on the survival of EcO157 and both are negatively correlated with *ttd*. NH_4_^+^-N, clay, and TP indirectly affected *ttd* by influencing the bacterial community structure. Clay and NH_4_^+^-N show positive correlation with bacterial community structure and TP shows negative correlation with bacterial community structure. As seen in Figure 6, NH_4_^+^-N and clay are negatively correlated with EcO157 survival, while TP is positively correlated with the survival of EcO157. The pH is both directly correlated with *ttd* and indirectly linked to *ttd* via the bacterial community.

## 4. Discussion

Soil texture, as indicated by sand, silt, and clay contents, may promote the flourishing of some species while restraining others [36]. In the current study, clay displayed an indirect effect on EcO157 survival via the bacterial community. Clay is positively correlated with the bacterial community, which is negatively linked to *ttd*. The explanation for this is that clay minerals are able to secure the water content and provide binding sites for organic materials and elements required for the bacterial community, and the clay particles may also offer protection from desiccation, gas diffusion, toxic exogenous compounds, and predation by protozoa [37,38], while the effect of the bacterial community on *ttd* is largely dependent on its composition and structure, as discussed below.

A previous study found that the survival of EcO157 was positively correlated with microbial diversity in soils [39], which is in agreement with our findings. It seems that a minimum number of species is essential for ecosystem function and a larger number of other species are probably necessary for maintaining the stability of an ecosystem [40]. As a result, it is easier for EcO157 to survive in a stable ecosystem with an abundant energy source and less competition [41]. A previous study showed that EcO157 interacted with the local microbial communities and produced a negative correlation, which is identical with our outcomes [42]. The adverse effect of the bacterial community on EcO157 survival is believed to be the result of predation, substrate competition, and antagonism [43].

Furthermore, we found that *Proteobacteria* was positively correlated with the survival of EcO157 while *Acidobacteria* was negatively correlated with the survival of EcO157. The relative abundance of *β-Proteobacteria* is most strongly related to carbon availability, and a higher relative abundance of *Proteobacteria* might imply more carbon in soils, which is essential for pathogen survival [25]. The *α-Proteobacteria* may contribute to Fe^2+^ production, which is necessary for pathogen survival [44]. As for *Acidobacteria*, previous analysis indicated that the response of the different subgroups of *Acidobacteria* to soil pH was varied. The relative abundance of subgroups 1, 2, 3, 12, 13, and 15 were negatively correlated with soil pH, whereas subgroups 4, 6, 7, 10, 11, 16, 17, 18, 22, and 25 were positively correlated with soil pH [45]. We assumed that in our soil samples, the subgroups of *Acidobacteria—*which were positively associated with pH—were more dominant, resulting in a negative correlation between *Acidobacteria* and *ttd*. Furthermore, other researchers believe that the relative abundance of *Acidobacteria* is negatively related to soil carbon availability [46,47]. Thus, a lower abundance of *Acidobacteria* equals to a greater carbon content, and therefore, survival is promoted.

The pH is one of the major factors influencing bacterial community structures [23]; it may impose physiological constraints on soil bacteria, alter competitive outcomes, or reduce the net growth of individual taxa [25]. A previous study revealed that the relative abundance of *δ-Proteobacteria* decreased as pH increased, but *α-Proteobacteria* increased [36]. In the current study, it was found that pH was an important factor influencing the survival of EcO157 and was negatively associated with *ttd* (Table 1, Figure 6). On the one hand, changes in pH can restrain the survival of EcO157 directly [48,49], although a previous study showed that EcO157 could survive longer in lower pH soils. Any change in the soil characteristics might alter soil pH [50], i.e., soil characteristics might vary a lot in soils with different pH values, and as a result, pH and other soil properties might display a complex influence on the survival of EcO157 [25,51]. In neutral and alkaline soils, EcO157 lost its preferred acidic environment, resulting in decreased viability. Notably, EcO157 was rapidly inactivated in strong alkaline soils, with a reduction in *ttd* to about 20 days, which was significantly lower than the time period observed in the other three group soils. Therefore, it was concluded that *ttd* and pH are negatively correlated.

NO_3_^−^-N was another key factor affecting *ttd* and it was negatively linked to *ttd* (Table 1, Figure 6). Generally, NO_3_^−^-N can provide nutrition for pathogens, and it may promote the survival of EcO157 [52]. Instead, our results showed that NO_3_^−^-N was negatively correlated with the survival of EcO157, and this could potentially be explained by the specific characteristics of soils from western Jilin Province. Due to ineffective management, excessive fertilization, and great differences in seasonal precipitation, soils in western Jilin Province suffered from salinization, exhibiting extreme soil physicochemical properties like high content of NO_3_^−^-N and high pH [14,53]. In this aspect, the inhibiting effect of NO_3_^−^-N on *ttd* is coincidental with the fact that a high pH would shorten *ttd*. In addition, we found that soils from central and eastern Jilin Province might support the survival of EcO157 by providing a moderate amount of NO_3_^−^-N as a nutrient substance. However, the resistance effect of basic soils was so strong that NO_3_^−^-N presented a negative correlation on the whole. The nitrogen sources in the soil most likely originate primarily from fertilization.. Some researchers have found that the influence of soil fertilization is closely related to soil pH variation (generally increases). The survival of EcO157 was significantly increased in acidic soils when an N source was applied. However, the promotion effect of the N source on the survival of EcO157 was greatly reduced in alkaline soil, which is consistent with the conclusion of our study. Experiments showed that when compared with chicken manure, pig manure application in soils has a more positive effect on the survival of EcO157 [54]. As mentioned above, NO_3_^−^-N may indirectly affect EcO157 by affecting the survival of other microorganisms in the soil. Appropriate inputs of an N source in soil may be helpful in maintaining the stability of the soil microbial community, which will hinder the invasion and survival of EcO157. Therefore, excessive fertilization behavior can lead to excessive NO_3_^−^-N content in soil, which can inhibit the survival of EcO157.

In addition, *ttd* correlates with other soil physicochemical properties like WSOC, NH_4_^+^-N, and EC. Soil WSOC and NH_4_^+^-N can guarantee sufficient energy sources and adjust soil–water content to provide a favorable environment for EcO157 persistence [55]. Consequently, the competitive pressure between indigenous microorganisms is decreased for pathogen survival [18,56]. Other researchers found that EC might influence the survival of EcO157 by a general osmotic effect or by specific ion toxicity, which leads to a decrease in activity in some enzymes that are essential for cell metabolism, e.g., K^+^ transport [7]. Furthermore, it is also possible that geographic location is responsible for the majority of alterations in the microbial community structure that impact the survival of EcO157. Previous research has indicated that soils situated at greater distances exhibited reduced similarity, implying that the number of species shared between soils sampled at greater distances was lower. Investigations into large-scale bacterial biogeography have demonstrated that bacterial community structure did indeed undergo changes with increasing geographical distance.

It should be noted that in the current study, we only tested one inoculation size of EcO157, but it would be interesting to investigate the effect of inoculation size on indigenous microbial communities. Such a goal could be achieved either by changing actual EcO157 inoculation size or by varying the concentrations of indigenous microbial communities as reported by Xing et al. (2020) [57]. Obtaining the genomic sequence of the mutant strain will lead to better characterization of its properties. Although this strain is associated with the primary pathogen, it may harbor mutations that could influence the results. In the current study, we only correlated the survival parameters (*ttd*, *p*, and *δ*) with soil properties (physicochemical properties and bacterial communities), characterized just before EcO157 inoculation. However, it is necessary to investigate the dynamic change in soil microbial communities during the survival study as performe by Xing et al., 2021 [58]. Furthermore, fungi present in the environment can continue to be incorporated into analytical studies as factors influencing the survival of EcO157. It also should be mentioned that it will be valuable to reveal the survival behavior of EcO157 when cell count falls below detection limit or when the cells enter into viable but non-culturable conditions (VBNCs); however, plate count protocol is not applicable in attempting to achieve this goal, and the next best available method is quantitative fluorescence PCR as reported previously [39].

## 5. Conclusions

The current research revealed that pH is an important factor that influences the survival of EcO157 in soils. pH not only directly correlates with the survival of EcO157 but also indirectly correlates with its survival by influencing other soil physicochemical properties and the bacterial community. In addition, NO_3_^−^-N is also an important influencing factor. Biotic factors like the composition and structure of the bacterial community played a crucial role in shaping the survival pattern of EcO157. Among these factors, the bacterial community was affected by several soil physicochemical properties, such as pH, clay, NH_4_^+^-N, and TP. In conclusion, the survival of EcO157 differed greatly in different areas over a pH gradient, and it expressed a weak persistence in basic soils compared to acidic and neutral soils. This research might help us better understand the survival activity of EcO157 and prevent infection outbreaks associated with this pathogen.

## Figures and Tables

**Table 1:** Stepwise multiple regression equation predicting *ttd* (time needed to reach detection limit) using soil properties and relative abundances of dominant phyla.

Regression Equation	*R* ^2^	*F*	*p*	*r* and *T* Value of Partial Regression Coefficient		
					*r*	*T*
*ttd* = −177.079 − 2.845 × pH − 3.912 × NO_3_^−^-N + 25.409 × *Proteobacteria* − 111.826 × *Acidobacteria* + 28.432 × *H*	0.861	8.155	0.000	pH NO_3_^−^-N *Proteobacteria* *Acidobacteria* *H*	−0.152 −0.274 0.074 −0.396 +0.825	−0.430 ** −1.629 ** −0.335 ** −1.136 ** +3.748 **

** Denotes significance at 0.01 level. pH, potential of hydrogen; NO_3_^−^-N, nitrate nitrogen; *Proteobacteria*, the relative abundance of *Proteobacteria*; *Acidobacteria*, the relative abundance of *Acidobacteria*; *H*, Shannon–Wiener index.

**Table 2:** Mantel and Partial Mantel tests between *ttd* (time needed to reach detection limit) and environment factors.

	Mantel	Partial Mantel
*H*	***	***
Bacterial community	***	***
NO_3_^−^-N	***	**
pH	***	*
*Acidobacteria*	***	-
WSOC	***	-
EC	**	-
*Proteobacteria*	**	-
NH_4_^+^-N	*	-
*Actinobacteria*	*	-

***, **, and * denotes significance at 0.001, 0.01, and 0.05, respectively.-denotes no significance. *H*, Shannon–Wiener index; bacterial community, the first axis scores from non-metric multidimensional scaling (NMDS); NO_3_^−^-N, nitrate nitrogen; pH, potential of hydrogen; *Acidobacteria*, the relative abundance of *Acidobacteria*; WSOC, water-soluble organic carbon; EC, electrical conductivity salinity; *Proteobacteria*, the relative abundance of *Proteobacteria*; NH_4_^+^-N, ammonium nitrogen; *Actinobacteria*, the relative abundance of *Actinobacteria.*

## Data Availability

The sequencing data have been deposited with links to BioProject under accession number PRJNA530627 for the bacterial community in the NCBI BioProject database. The original contributions presented in this study are included in the article. Further inquiries can be directed to the corresponding author.

## Supplementary Materials

The following supporting information can be downloaded at https://www.mdpi.com/article/10.3390/microorganisms13112492/s1. Figure S1: Bacterial community composition (abundance > 1%) at phylum level in weak acidic, neutral, weak basic, and strong basic soils, respectively; Figure S2: Abundance of dominant phyla in 24 soil samples(abundance > 1%); Figure S3: Bacterial community composition (abundance > 1%) at class level in weak acidic, neutral, weak basic, and strong basic soils, respectively; Figure S4: NMDS analysis of soil bacterial communities (T1–T8: weak acidic soils, T9–T13: neutral soils, T14–T18: weak basic soils, T19–T24: strong basic soils); Figure S5: Canonical correlation analysis (CCA) among soil physicochemical properties, microbial communities and survival parameters; Table S1: Latitude and longitude coordinates of sampling points; Table S2: Soil properties of 24 samples. EC, electrical conductivity, NH_4_^+^-N, ammonium nitrogen, NO_3_^−^-N, nitrate nitrogen, WSOC, water-soluble organic carbon, TN, soluble total nitrogen, TP, total phosphorus; Table S3: Richness index of soil microbial community; Table S4: Difference analysis of β diversity of bacterial community among different pH gradient (Adonis); Table S5: Co-occurrence network topological parameters.
