# Supplementary material for: Survival of Escherichia coli O157:H7 in Soils Along a Natural pH Gradient
Source: Microorganisms. 2025 Oct 30;13(11):2492. doi: 10.3390/microorganisms13112492 (PMC12654261; doi:10.3390/microorganisms13112492)
Supplement: Supplementary file 1 [file microorganisms-13-02492-s001.zip › microorganisms-3932334-supplementary.pdf]

Supporting Information

Survival of *Escherichia coli* O157:H7 in soils along a natural pH gradient

Guangze Lyu[1,2], Huiru Li[2], Jiayang Hu [2], Jincai Ma[2*]

1. Key Laboratory of Ground Water Resource and Environment, Ministry of Education, Jilin University, Changchun 130021, China
2. Jilin Provincial Key Laboratory of Water Resources and Environment, Jilin University, Changchun 130021, China

* Correspondence author
Dr. Jincai Ma
E-mail: jincaima@jlu.edu.cn
Phone: +86-431-85168429
College of New Energy and Environment
Jilin University

Table S1. Latitude and longitude coordinates of sampling points

Samples	Longitude	Latitude
T1	127°19'34"	43°50'6"
T2	126°55'49"	43°52'1"
T3	127°17'39"	44°0'48"
T4	127°13'44"	44°10'18"
T5	126°47'39"	44°16'2"
T6	126°35'14"	44°8'45"
T7	126°11'33"	43°53'58"
T8	126°27'10"	44°35'4"
T9	124°59'51"	45°1'48"
T10	125°27'50"	44°29'13"
T11	126°24'9"	44°57'5"
T12	124°47'27"	44°40'32"
T13	124°36'51"	44°46'24"
T14	124°23'41"	45°22'1"
T15	124°34'49"	45°15'5"
T16	124°39'12"	45°9'17"
T17	125°5'1"	44°45'42"
T18	125°22'47"	44°38'1"
T19	123°41'32"	45°8'42"
T20	123°36'31"	45°21'22"
T21	123°59'12"	45°25'53"
T22	123°53'6"	45°28'22"
T23	123°45'43"	45°37'8"
T24	123°33'1"	45°38'12"

Table S2. Soil properties of 24 samples. EC, electrical conductivity, NH_4^+-N, ammonium nitrogen, NO_3^--N, nitrate nitrogen, WSOC, water soluble organic carbon, TN, soluble total nitrogen, TP, total phosphorus.

Samples	pH	EC (mS/cm)	NH_4^+-N (mg/kg)	NO_3^--N (mg/kg)	TP (mg/kg)	WSOC (mg/kg)	Clay （%）
T1	5.61	0.72	1.21	6.80	6.30	94.15	4.21
T2	5.70	0.17	1.18	13.70	2.70	97.35	4.93
T3	5.75	0.04	2.15	5.86	8.90	190.75	5.37
T4	5.76	0.11	0.77	6.14	23.00	58.10	6.49
T5	5.82	0.06	1.41	5.08	3.05	26.65	6.82
T6	5.82	0.07	1.11	7.82	2.30	96.88	7.88
T7	6.30	0.17	2.59	28.90	30.00	203.75	8.33
T8	6.40	0.19	1.41	1.06	19.00	137.20	8.09
T9	6.81	1.20	1.21	0.12	17.00	171.83	6.03
T10	7.08	0.11	0.76	2.28	25.50	111.20	6.89
T11	7.28	0.17	1.46	6.11	5.75	162.45	6.98
T12	7.36	0.07	1.73	6.34	5.55	133.10	2.80
T13	7.43	0.08	2.82	8.92	3.15	158.50	2.88
T14	7.52	0.06	1.65	1.52	2.80	92.08	4.62
T15	7.55	0.16	1.25	5.43	2.05	201.50	5.39
T16	7.61	0.09	0.63	2.64	4.65	124.83	5.26
T17	7.72	0.62	3.33	16.71	26.05	231.03	6.98
T18	8.07	0.17	16.25	7.18	3.80	188.20	7.01
T19	9.26	8.57	0.55	240.02	8.08	658.13	6.50
T20	9.56	0.47	1.62	60.07	3.95	278.90	3.11
T21	9.67	0.99	10.92	65.58	18.05	855.98	21.07
T22	9.79	1.16	1.61	27.19	7.82	91.13	6.28
T23	9.8	0.81	17.31	0.70	8.41	454.15	6.54
T24	9.92	1.49	40.79	130.58	36.10	2182.40	9.91

Table S3. Richness index of soil microbial community

Samples	Chao1	ACE	Shannon-Wiener
T1	2693.87	2775.29	10.01
T2	4212.60	4134.77	10.56
T3	2476.63	2565.84	9.67
T4	2526.84	2616.55	9.78
T5	2812.60	2791.39	9.95
T6	2543.12	2558.00	9.93
T7	3570.60	3557.71	10.39
T8	3846.42	3707.87	10.33
T9	2989.92	3043.84	10.18
T10	2567.00	2567.00	10.16
T11	3942.81	3964.02	10.63
T12	4250.41	4257.77	10.79
T13	3820.63	3770.85	10.44
T14	2497.00	2497.00	9.96
T15	2823.80	2839.49	10.50
T16	3563.14	3697.15	10.44
T17	2518.00	2518.00	10.21
T18	3641.06	3851.51	10.51
T19	1004.00	1004.70	8.47
T20	1778.62	1799.96	8.78
T21	1520.03	1522.30	9.10
T22	1129.00	1129.00	8.26
T23	1313.51	1320.78	8.40
T24	1512.67	1560.17	8.35

Table S4. Difference analysis of β diversity of bacterial community among different pH gradient (Adonis)

		Over all	Weak acidic-neutral	Weak acidic-weak basic	Weak acidic-strong basic	Weak basic-neutral	Weak basic-strong basic	Neutral-strong basic
bray	*F*	38.78***	24.34*	52.61***	46.94***	19.57	55.29***	54.14***
jaccard	*F*	5.66***	5.78*	6.60***	4.64***	4.31	6.93***	5.66***

Note: In the table, * indicates significant correlation, * indicates $P<0.05$, ** indicates $P<0.01$, and *** indicates $P<0.001$

Table S5. Co-occurrence network topological parameters

Network metrics	Nodes	Edges	Average Degree	Average Weighted Degree	Modularity	Average Clustering Coefficient	Average Path Length
values	17	87	7.817	4.126	0.487	0.515	2.245

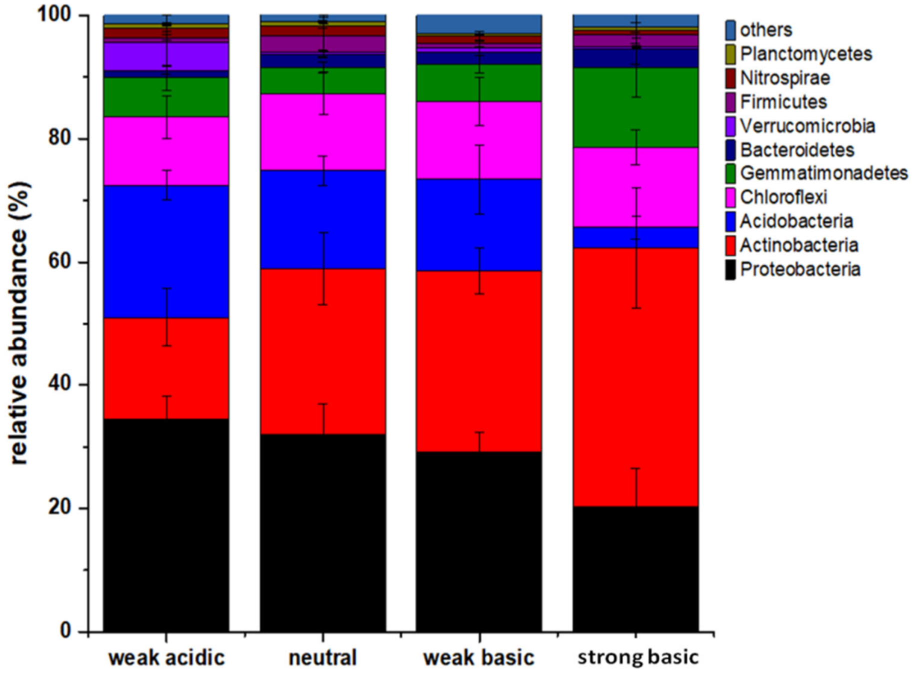

Figure S1. Bacterial community composition (abundance >1%) at phylum level in weak acidic, neutral, weak basic, and strong basic soils, respectively.

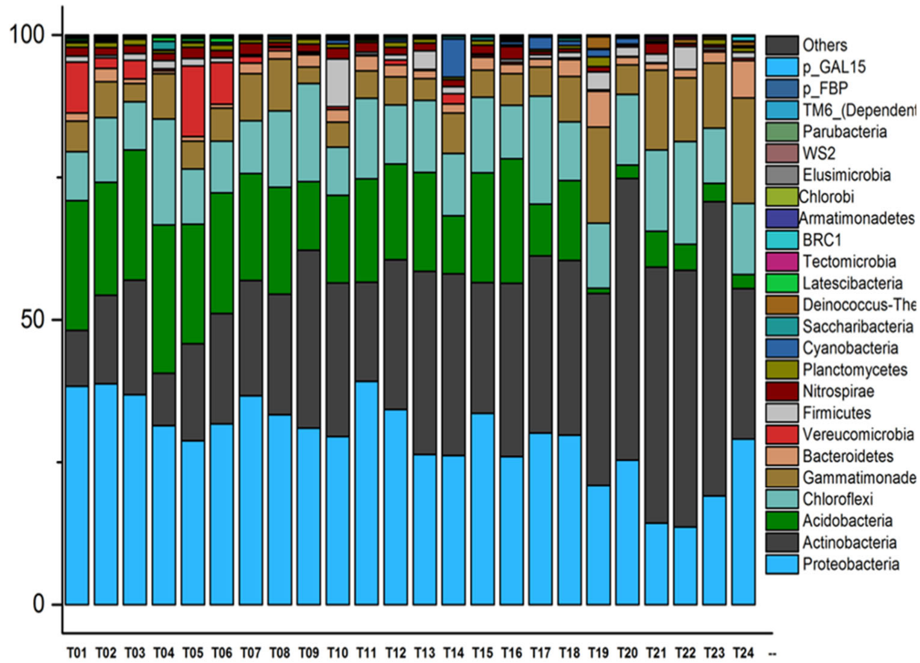

Figure S2. Abundance of dominant phyla in 24 soil samples(abundance >1%).

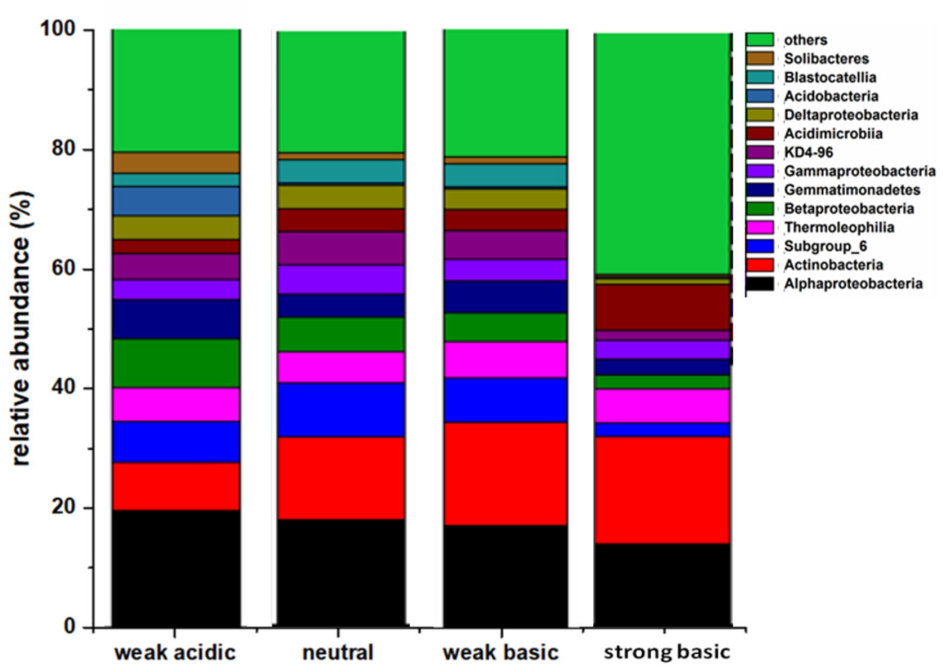

Figure S3. Bacterial community composition (abundance >1%) at class level in weak acidic, neutral, weak basic, and strong basic soils, respectively.

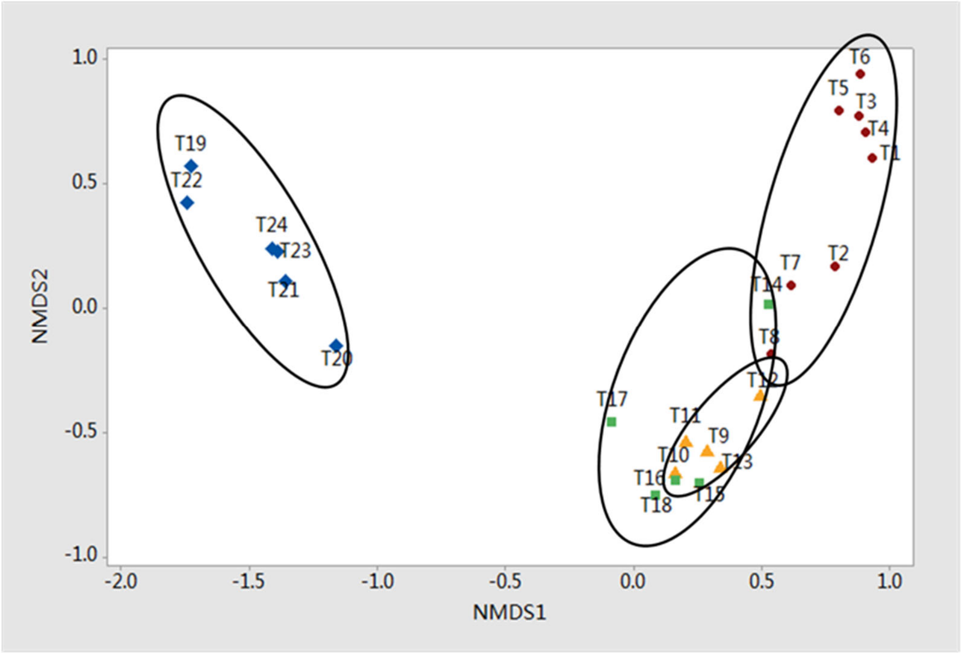

Figure S4. NMDS analysis of soil bacterial communities(T1-T8: weak acidic soils, T9-T13: neutral soils, T14-T18: weak basic soils, T19-T24: strong basic soils)

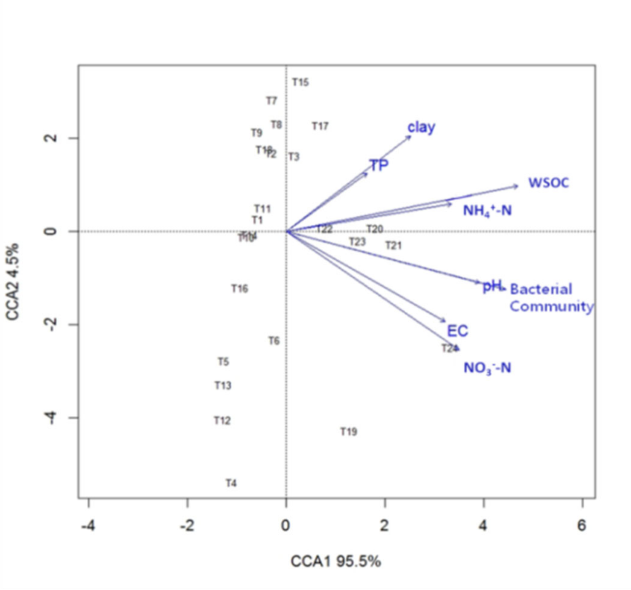

Figure S5. Canonical correlation analysis(CCA) among soil physicochemical properties, microbial communities and survival parameters
